# Trends in the surgical procedures of women with incident breast cancer in Catalonia, Spain, over a 7-year period (2005–2011)

**DOI:** 10.1186/1756-0500-7-587

**Published:** 2014-09-01

**Authors:** Josep M Escribà, Laura Pareja, Laura Esteban, Jordi Gálvez, Angels Melià, Laura Roca, Ramon Clèries, Xavier Sanz, Montse Bustins, María J Pla, Miguel J Gil, Josep M Borrás, Josepa Ribes

**Affiliations:** Catalonian Cancer Registry, Cancer Planning Directorate, Av. Gran Vía 199-203, 08908 L’Hospitalet de Llobregat, Spain; Department of Clinical Sciences, University of Barcelona, Bellvitge Campus, L’Hospitalet de Llobregat, Kragujevac, Spain; Servei d’ Informació i Estudis, Catalonian Health Service, Health Department, Generalitat de Catalunya, L’Hospitalet de Llobregat, Kragujevac, Spain; Breast Cancer Functional Unit, Institut Català d’ Oncologia, L’Hospitalet de Llobregat, Kragujevac, Spain

**Keywords:** Breast cancer, Incident cases, Hospital discharge dataset, Surgical procedures

## Abstract

**Background:**

Breast cancer (BC) is the most frequent cancer in women, accounting for 28% of all tumors among women in Catalonia (Spain). Mastectomy has been replaced over time by breast-conserving surgery (BCS) although not as rapidly as might be expected. The aim of this study was to assess the evolution of surgical procedures in incident BC cases in Catalonia between 2005 and 2011, and to analyze variations based on patient and hospital characteristics.

**Methods:**

We processed data from the Catalonian Health Service’s Acute Hospital Discharge database (HDD) using ASEDAT software (Analysis, Selection and Extraction of Tumor Data) to identify all invasive BC incident cases according to the codes 174.0-174.9 of the International Classification of Diseases, Ninth Revision, Clinical Modification (ICD-9-CM) that were attended for the one-year periods in 2005, 2008 and 2011. Patients were classified according to surgical procedures (BCS vs mastectomy, and immediate vs delayed reconstruction), and results were compared among periods according to age, stage, comorbidity and hospital level.

**Results:**

BC surgical procedures were performed in more than 80% of patients. Surgical cases showed a significant increasing trend in the proportion of women aged 50–69 years, more advanced disease stages, higher comorbidity and they were attended in hospitals of less complexity level throughout the study period. Similar pattern was found for patients treated with BCS, which increased significantly from 67.9% in 2005 to 74.0% in 2011.

Simple lymph node removal increased significantly (from 48.8% to 71.4% and from 63.6% to 67.8% for 2005 and 2011 in conservative and radical surgery, respectively). A slightly increase in the proportion of mastectomized young women (from 28% in 2005 to 34% in 2011) was detected, due to multiple factors. About 22% of women underwent post-mastectomy breast reconstruction, this being mostly immediate.

**Conclusions:**

The use of HDD linked to the ASEDAT allowed us to evaluate BC surgical treatment in Catalonia. A consolidating increasing trend of BCS was observed in women aged 50–69 years, which corresponds with the pattern in most European countries. Among the mastectomized patients, immediate breast reconstructions have risen significantly over the period 2005–2011.

**Electronic supplementary material:**

The online version of this article (doi:10.1186/1756-0500-7-587) contains supplementary material, which is available to authorized users.

## Background

Breast cancer (BC) remains one of the most common tumors in western countries, accounting for around 25-30% of cancers among women [1], in addition to being one of the leading causes of death in these populations. The steady rise in incidence over the past decades is attributable to the increased prevalence of certain risk factors (such as hormone replacement therapy in menopause) and the aging population. Improved diagnostic capabilities, in the form of mammography screening, also account for a spike in incidence [2, 3].

In Spain, the incidence in women over 45 years has stabilized since 2001 due to screening saturation; this phenomenon is clearly observed where breast screening programs were implemented before the year 2000 and had high participation rates [4]. Among younger women, the incidence is increasing, probably reflecting lifestyle changes in these generations [5]. On the other hand, mortality has declined since the 1990s mainly due to earlier detection of tumors and therapeutic advances [6, 7].

In 2007, the age-adjusted incidence and mortality rates in Catalonia (67.5 and 11.1 per 10^5^ person-years, respectively) [8] were below the European Union average (77.1 and 16.6 cases per 10^5^ person-years, respectively), but above the Spanish average (61.0 and 12.9 per 10^5^ person-years, respectively). As in the rest of the country, however, indicators have improved: incidence has stabilized, and mortality has decreased by 3.7% a year since the early 1990s. The five-year relative survival rate was 81% for women diagnosed in 1995–1999, in comparison to 76% for the 1990–1994 period [9].

Surgical resection remains the main treatment for BC. However, in recent years there have been significant advances in breast-conserving surgery (BCS), which have optimized treatment success when combined with radiation and hormone therapy. As a result of this and early detection, a large proportion of women have been able to benefit from minimally invasive surgical techniques for the breast and axilla since the turn of the century. Monitoring of surgical procedures among breast cancer patients would allow public health authorities to evaluate the implementation of cancer clinical practice guidelines and the quality of treatments carried out in hospitals [10].

The present study examines the surgical procedures for incident cases of BC that were diagnosed over the three, one-year periods in Catalonia (2005, 2008 and 2011), using the regional population-based database of hospital discharges. Data were analyzed according to socio-demographic (hospital level) and clinical (age, comorbidity and clinical stage) characteristics, with the aim of defining dictionaries and algorithms enabling us to automate the collection of indicators.

## Methods

### Data source

We used the Acute Hospital Discharge Dataset (HDD), which includes records for all inpatient admissions from public and private hospitals in Catalonia. This database includes all episodes of healthcare in Catalonian health centers that led to patient admission into modalities including conventional hospitalization, major same-day surgery, outpatient day hospital and domiciliary hospitalization [11]. Data for the 2003–2012 time period were made available by the Catalonian Health Service, Health Department of the Catalonian Government.

From the HDD, BC cases were identified on the basis of the International Classification of Diseases, Ninth Revision, Clinical Modification (ICD-9-CM) through the ASEDAT software [12]. ASEDAT was developed following the International Agency for Research on Cancer (IARC) recommendations [13]. Based on computer algorithms, it was designed to automatically detect and extract incident cancer cases from different databases, including the HDD in a given study period, with the purpose of increasing data reliability and reduce costs associated with a cancer registry.

We included women diagnosed with invasive breast cancer (i.e., malignant neoplasm of female breast; ICD-9-CM codes: 174.0-174.9) [14]. Data related to patients with in situ breast carcinoma (ICD-9-CM code 233.0) were excluded.

### Identifying hospital episodes of care for breast cancer patients

Administrative hospital discharge abstracts from these BC cases were used to appropriately identify and construct the complete hospital episodes belonging to each patient. Patient abstracts were sorted in ascending order according to date of admission and discharge date, in that order.

Hospital episodes were defined by grouping together consecutive abstracts that were closely related in time (i.e., <24 hrs between them) and involved in a continuous healthcare process such as inter-hospital and intra-hospital transfers.

Patients were identified by their valid Personal Health Identification Numbers (the social security number), which were used to match hospital abstracts with individual patients. These data were pulled from the Catalonian HDD from 2003 onwards, enabling us to identify and compile all the different public hospital discharges where an individual had been attended. The result for each patient was one or more hospital episodes, each constructed from the corresponding hospital abstracts.

Episodes of inpatient care attended in private health centers were excluded from the analysis, because private hospitals do not use the Personal Health Identification Number system-wide, and we were unable to identify them within the public health system. Prevalent cases, defined as those with evidence of breast disease prior to the respective study periods, were also excluded by ASEDAT.

### Defining the population and the breast cancer procedures carried out

In summary, we used the Catalonian HDD to identify all women with incident invasive BC diagnosed and attended during the years 2005, 2008 and 2011.

We only examined three one-year periods because of: 1) about 40% cases (1200 cases per year) remained unsolved after applying ASEDAT for the whole period, and they need to be manually checked by reviewing computer medical records, making it unfeasible; 2) the reported years are equidistants; and 3) data available for the time period 2003–2012 allow us to discard prevalent cases and ensure the minimum 12-month follow-up for incident BC cases.

For each woman, an index surgical procedure (BCS or mastectomy) was identified, as were subsequent surgical procedures related to breast reconstruction that took place within 12 months. Primary BCS and radical surgery procedures were identified based on the ICD-9-CM. Likewise, we also considered other related procedures such as removal of lymphatic structures (simple or radical), and reconstruction (immediate or delayed) (Additional file 1: Table S1). Immediate breast reconstruction was defined as the presence of reconstructive surgery either as a primary or as a secondary procedure that took place simultaneously within the same first surgical episode as the mastectomy. Delayed reconstruction was limited to procedures performed within 12 months after the mastectomy. According to the ICD-9-CM codes used, our patients did not undergo any procedures of BCS or mastectomy during the three one-year study periods were classified as non-surgical.

Hospitals were categorized into three groups according to the Catalonian Health Service classification: i) high technology, including health centers providing highly specialized services, ii) reference hospitals and iii) county hospitals. In addition, the Charlson Comorbidity Index (CCI) [15], was calculated taking into account all the main and secondary diagnoses for the previous and reference principal episode.

Results were analyzed according to (a) age groups (≤49, 50–69 and ≥70 years old); (b) stage of disease at diagnosis (using ICD-9-CM coding as a proxy) [16, 17] (Additional file 1: Table S2a); (c) hospital complexity; and (d) comorbidity. Three levels of comorbidity were defined according to Quan’s update of the CCI, as applied to ICD-9-CM [18]: no comorbidity = 0, low comorbidity = from 1 to median truncated to 1, and high comorbidity = more than the median truncated to 1.

Tumor stage was classified as local (confined within the breast), regional (affecting the lymph nodes, primarily those in the armpit and/or upper arm) or distant (the cancer is found in other parts of the body as well). Hospital discharge ICD-9 codes were mapped using the American Joint Committee on Cancer (AJCC) stages [17] (Additional file 1: Tables S2a and S2b).

### Statistical analysis

For each variable considered in the analysis, trends in proportions during the years 2005, 2008 and 2011 were assessed using a log-binomial regression model [19]. The sign of the slope of this model (β) indicates if there was an increasing (positive) or decreasing (negative) trend in the proportions [19]. Statistical analyses were carried out through the statistical software R [20] assuming a significance level of α = 0.05.

### Ethics

The study uses retrospective data from administrative databases in where patients are anonymus to the researchers and did not require informed consent nor Ethics Committee approval.

## Results

As shown in Table 1, the number of patients identified with incident BC in 2005, 2008 and 2011 were 3739, 3849 and 3873 respectively, of whom 2971 (79.5%), 3218 (83.6%) and 3330 (86%) underwent surgery in the Catalonian public health system. It is worth noting that there were statistically significant increases in the proportions of BC surgically treated.

**Table 1:** **Characteristics of breast cancer patients diagnosed in 2005, 2008 and 2011 in Catalonia, Spain, according to surgical treatment**

	SURGERY							NO SURGERY						
	2005		2008		2011			2005		2008		2011		
	n	(%)	n	(%)	n	(%)	β* (95% CI)	n	(%)	n	(%)	n	(%)	β* (95% CI)
**Age**														
≤49	795	(26.8)	915	(28.4)	876	(26.3)	-0.003 (-0.017; 0.010)	119	(15.5)	101	(16.0)	83	(15.3)	-0.001 (-0.044; 0.041)
50 - 69	1344	(45.2)	1511	(47.0)	1642	(49.3)	**0.014 (0.006; 0.023)**	266	(34.6)	215	(34.1)	182	(33.5)	-0.005 (-0.031; 0.020)
≥ 70	832	(28.0)	792	(24.6)	812	(24.4)	**-0.023 (-0.037;-0.009**)	383	(49.9)	315	(49.9)	278	(51.2)	0.004 (-0.014; 0.022)
**Stage**														
Local	2713	(91.3)	2794	(86.8)	2685	(80.6)	**-0.020 (-0.023;-0.017)**	474	(61.7)	370	(58.6)	328	(60.4)	-0.004 (-0.020; 0.010)
Regional	226	(7.6)	381	(11.8)	582	(17.5)	**0.137 (0.114; 0.160)**	24	(3.1)	14	(2.2)	15	(2.8)	-0.027 (-0.140; 0.081)
Distant	32	(1.1)	43	(1.3)	63	(1.9)	**0.096 (0.027; 0.167)**	270	(35.2)	247	(39.1)	200	(36.8)	0.009 (-0.015; 0.032)
**Charlson Comorbidity Index**														
No comorbidity	2490	(83.8)	2651	(82.4)	2674	(80.3)	**-0.007 (-0.011;-0.003)**	545	(71.0)	405	(64.2)	339	(62.4)	**-0.023 (-0.036; -0.010)**
Low comorbidity	375	(12.6)	440	(13.7)	485	(14.6)	**0.024 (0.003; 0.045)**	135	(17.6)	119	(18.9)	113	(20.8)	0.028 (-0.010; 0.065)
High comorbidity	106	(3.6)	127	(3.9)	171	(5.1)	**0.063 (0.023; 0.103)**	88	(11.5)	107	(17.0)	91	(16.8)	**0.062 (0.019; 0.105)**
**Hospital complexity**														
Low	481	(16.2)	560	(17.4)	619	(18.6)	**0.023 (0.005; 0.041)**	196	(25.5)	149	(23.6)	133	(24.5)	-0.008 (-0.040; 0.024)
Medium	1297	(43.7)	440	(45.3)	485	(48.4)	**-0.024 (-0.240;-0.207)**	341	(44.4)	284	(45.0)	252	(46.4)	0.007 (-0.013; 0.027)
High	1193	(40.2)	127	(37.3)	171	(33.0)	**-0.468 (-0.496;-0.440)**	231	(30.1)	198	(31.4)	158	(29.1)	-0.004 (-0.032; 0.023)
**Emergency admission**	85	(2.9)	104	(3.2)	101	(3.0)	0.009 (-0.038;0.055)	388	(50.5)	379	(60.1)	334	(61.5)	**0.032 (0.017; 0.048)**
**In-hospital deaths** ^**1**^	5	(0.2)	1	(0.0)	3	(0.1)	-0.133 (-0.429;0.137)	80	(10.4)	80	(12.7)	59	(10.9)	0.010 (-0.041; 0.061)
**Discharge destination**														
Domicile	2951	(99.3)	3200	(99.4)	3299	(99.1)	0 (-0.001;0.001)	659	(85.8)	496	(78.6)	414	(76.2)	**-0.021 (-0.031;-0.012)**
With support	17	(0.6)	15	(0.5)	30	(0.9)	0.089 (-0.015;0.195)	43	(5.6)	61	(9.7)	77	(14.2)	**0.152 (0.095; 0.210)**
Other^2^	3	(0.1)	3	(0.1)	1	(0.0)	-0.168 (-0.520;0.141)	66	(8.6)	74	(11.7)	52	(9.6)	0.021 (-0.033; 0.076)
**Total** ^**3**^	**2971**	**(79.5)**	**3218**	**(83.6)**	**3330**	**(86.0)**	**0.013 (0.009; 0.016)**	**768**	**(20.5)**	**631**	**(16.4)**	**543**	**(14.0)**	**-0.064 (-0.081;-0.048)**

*β is the slope of the log-binomial model used to measure the temporal trend; 95% CI: 95% Confidence Interval; In bold, statistically significant;

^1^Mortality within 30 days after admission; ^2^Includes hospital mortality during hospitalization (in-hospital mortality); ^3^Percentages were calculated taking into account the total number of patients (surgical and non-surgical) in the respective years.

Over the years, we found a significant increase of proportion of women aged 50–69 among surgical cases (from 45.2% in 2005 to 47.0% in 2008 and finally 49.3% in 2011). There were also increases in the percentage of patients presenting with regional tumor stage (7.6%, 11.8% and 17.5% respectively) and distant tumor stage at diagnosis (1.1%, 1.3% and 1.9%). The proportion of patients who presented with comorbid diseases increased among those with both low comorbidity (12.6%, 13.7% and 14.6%) and high comorbidity (3.6%, 3.9% and 5.1%) (Table 1).

The percentage of BC patients who did not undergo surgery is around 17% for the entire period. Regardless of the year, non-surgical compared to surgical patients showed more advanced disease at diagnosis, greater comorbidity, more frequent admittance through the emergency department and in low complexity health centers (county hospitals), more home healthcare requirements, and higher 30-day mortality (Table 1).

### Conservative or radical surgery

In 2011 in Catalonia, BCS accounted for 74% of BC surgery, a significant increase in comparison to 2005 and 2008. BCS use was increasing in the 50–69 age group (49.8%, 52.7% and 54.5% in 2005, 2008 and 2011, respectively) and among women with presenting with regional tumor stage (7.6%, 10.7% and 16.2%).

In high-tech hospitals, a significant decrease in the percentage of patients treated with BCS (40.5%, 36.1% and 32.6%) was observed, contrary to the trend observed in health centers of medium (44.2%, 45.8% and 48.4%) and low complexity (15.3% to 18.1% and 18.9%), where it significantly increased (Table 2).

**Table 2:** **Characteristics of breast cancer patients diagnosed in 2005, 2008 and 2011 in Catalonia, Spain, according to type of surgery**

	Conservative surgery							Radical surgery						
	2005		2008		2011			2005		2008		2011		
	n	(%)	n	(%)	n	(%)	β* (95% CI)	n	(%)	n	(%)	n	(%)	β* (95% CI)
**Age**														
≤49	528	(26.2)	614	(26.3)	583	(23.7)	**-0.017 (-0.034; -0.001**)	267	(28.0)	301	(34.0)	293	(33.8)	**0.030 (0.008;0.053)**
50- 69	1005	(49.8)	1229	(52.7)	1343	(54.5)	**0.015 (0.006; 0.024)**	339	(35.6)	282	(31.9)	299	(34.5)	-0.006 (-0.027; 0.016)
≥ 70	485	(24.0)	490	(21.0)	537	(21.8)	-0.016 (-0.034; 0.003)	347	(36.4)	302	(34.1)	275	(31.7)	**-0.023 (-0.044;-0.002)**
**Stage**													cp	
Local	1849	(91.6)	2072	(88.8)	2039	(82.8)	**-0.016 (-0.020; -0.013)**	864	(90.7)	722	(81.6)	646	(74.5)	**-0.033 (-0.040;-0.026)**
Regional	153	(7.6)	250	(10.7)	398	(16.2)	**0.128 (0.100; 0.157)**	73	(7.7)	131	(14.8)	184	(21.2)	**0.161 (0.122; 0.201)**
Distant	16	(0.8)	11	(0.5)	26	(1.1)	0.064 (-0.047; 0.179)	16	(1.7)	32	(3.6)	37	(4.3)	**0.141 (0.054; 0.230)**
**Charlson Comorbidity Index**														
No comorbidity	1718	(85.1)	1947	(83.5)	1995	(81.0)	**-0.008 (-0.013; -0.004)**	772	(81.0)	704	(79.5)	679	(78.3)	-0.006 (-0.013;0.002)
Low comorbidity	243	(12.0)	305	(13.1)	355	(14.4)	**0.030 (0.005; 0.055)**	132	(13.9)	135	(15.3)	130	(15.0)	0.013 (-0.024;0.050)
High comorbidity	57	(2.8)	81	(3.5)	113	(4.6)	**0.082 (0.031; 0.134)**	49	(5.1)	46	(5.2)	58	(6.7)	0.045 (-0.018;0.108)
**Type of surgery**														
Tumorectomy/Partial mastectomy	2018	(100.0)	2333	(100.0)	2463	(100.0)		-	-	-	-	-	-	
Simple mastectomy	-	-	-	-	-	-		812	(85.2)	757	(85.5)	719	(82.9)	-0.004 (-0.011;0.002)
Radical mastectomy	-	-	-	-	-	-		124	(13.0)	107	(12.1)	110	(12.7)	-0.005 (-0.045;0.306)
Subcutaneous mastectomy	-	-	-	-	-	-		17	(1.8)	21	(2.4)	38	(4.4)	**0.157 (0.064; 0.254)**
**Lymph node removal**	1556	(77.1)	1990	(85.3)	2122	(86.2)	**0.016 (0.012; 0.021)**	830	(87.1)	783	(88.5)	776	(89.5)	0.005 (-0.001;0.010)
**Type of lymph node removal** ^**1**^														
Simple	759	(48.8)	1349	(67.8)	1516	(71.4)	**0.070 (0.061; 0.079)**	528	(63.6)	559	(71.4)	526	(67.8)	**0.015 (0.002;0.027)**
Radical	797	(51.2)	641	(32.2)	606	(28.6)	**-0.082 (-0.097;-0.067)**	302	(36.4)	224	(28.6)	250	(32.2)	-0.018 (-0.042;0.007)
**Hospital complexity**														
Low	309	(15.3)	422	(18.1)	466	(18.9)	**0.034 (0.012; 0.055)**	172	(18.0)	138	(15.6)	153	(17.6)	-0.005 (-0.039;0.029)
Medium	892	(44.2)	1068	(45.8)	1193	(48.4)	**0.015 (0.005; 0.026)**	405	(42.5)	390	(44.1)	420	(48.4)	**0.022 (0.005;0.039)**
High	817	(40.5)	843	(36.1)	804	(32.6)	**-0.036 (-0.049;-0.023)**	376	(39.5)	357	(40.3)	294	(33.9)	**-0.023 (-0.043;-0.004)**
**In-hospital deaths** ^**2**^	1	(0.0)	0	(0.0)	2	(0.1)	0.144 (-0.355; 0.749)	4	(0.4)	1	(0.1)	1	(0.1)	-0.260 (-0.718; 0.088)
**Discharge destination**														
Domicile	2008	(99.5)	2328	(99.8)	2451	(99.5)	0 (-0.001; 0.001)	943	(99.0)	872	(98.5)	848	(97.8)	-0.002 (-0.004; 0.001)
With support	9	(0.4)	4	(0.2)	11	(0.4)	0.009 (-0.156; 0.178)	8	(0.8)	11	(1.2)	19	(2.2)	**0.164 (0.032;0.304)**
Other^3^	1	(0.0)	1	(0.0)	1	(0.0)	-0.033 (-0.535; 0.469)	2	(0.2)	2	(0.2)	0	(0.0)	-0.260 (-0.850;0.162)
**Total** ^**4**^	**2018**	**(67.9)**	**2333**	**(72.5)**	**2463**	**(74.0)**	**0.014 (0.009; 0.019)**	**953**	**(32.1)**	**885**	**(27.5)**	**867**	**(26.0)**	**-0.035 (-0.048;-0.022)**

*β is the slope of the log-binomial model used to measure the temporal trend; 95% CI: 95%Confidence Interval; In bold, statistically significant; ^1^Percentages were calculated taking into account the total number of lymph node removal; ^2^Mortality within 30 days after admission; ^3^Includes hospital mortality during hospitalization (in-hospital mortality); ^4^Percentages were calculated taking into account the total number of surgical patients in the respective years.

Likewise, a significant drop in radical surgery was observed in the same period (from 32.1% in 2005 to 26.0% in 2011), mainly in patients over 50 years old. On the other hand, we detected a significant rise in mastectomies in younger women (from 28.0% to 33.8%). According to the disease stage, there is a clear decline over time in the practice of mastectomy in cases of local disease (from 90.7% to 74.5%) and a significant increase for regional (from 7.7% to 21.2%) and metastatic tumors (from 1.7% to 4.3%). The most common modality of radical breast surgery was, by far, the simple mastectomy (around 85% of all mastectomies throughout the study period); radical mastectomy and subcutaneous mastectomy followed at a considerable distance (Table 2).

Lymph node surgery in BCS significantly increased (from 77.1% in 2005 to 86.2% in 2011), with important advances in simple lymph node dissection (from 48.8% to 71.4%). In mastectomized women, removal of lymph nodes remained stable throughout time at around 88%, and we detected a significant increase of simple lymph node excisions.

### Mastectomy and immediate reconstruction

Around 22% of mastectomized patients underwent breast reconstructive surgery in the Catalonian public health system (17.1%, 25.3% and 22.8% in 2005, 2008 and 2011, respectively). This percentage increased significantly in women with regional stage cancer (8.6%, 21.4% and 21.2% respectively), and in those who were intervened in reference hospitals (28.8%, 34.4% and 42.9%). Furthermore, the proportion of delayed reconstruction declined significantly (22.7%, 14.3% and 5.6%) (Table 3).

**Table 3:** **Mastectomized breast cancer patients with reconstructive surgery diagnosed in 2005, 2008 and 2011 in Catalonia, Spain**

	Radical surgery						
	2005		2008		2011		
	n	(%)	n	(%)	n	(%)	β* (95% CI)
**Age**							
≤49	99	(60.7)	147	(65.6)	121	(61.1)	0.000 (-0.026; 0.026)
50 - 69	56	(34.4)	68	(30.4)	69	(34.8)	0.004 (-0.045; 0.054)
≥ 70	8	(4.9)	9	(4.0)	8	(4.0)	-0.032 (-0.197; 0.132)
**Stage**							
Local	144	(88.3)	173	(77.2)	148	(74.7)	-**0.030 (-0.047;-0.012)**
Regional	14	(8.6)	48	(21.4)	42	(21.2)	**0.113 (0.038; 0.191)**
Distant	5	(3.1)	3	(1.3)	8	(4.0)	0.071 (-0.137; 0.290)
**Charlson Comorbidity Index**							
No comorbidity	143	(87.7)	200	(89.3)	177	(89.4)	0.003 (-0.009; 0.015)
Low comorbidity	15	(9.2)	21	(9.4)	18	(9.1)	-0.002 (-0.110; 0.107)
High comorbidity	5	(3.1)	3	(1.3)	3	(1.5)	-0.132 (-0.400; 0.118)
**Type of surgery**							
Simple mastectomy	129	(38.7)	175	(78.1)	149	(75.3)	-0.008 (-0.027; 0.010)
Radical mastectomy	17	(8.6)	30	(13.4)	13	(6.6)	-0.067 (-0.168; 0.034)
Subcutaneous mastectomy	17	(52.8)	19	(8.5)	36	(18.2)	**0.117 (0.021; 0.216)**
**Type of reconstruction**							
Immediate	126	(77.3)	192	(85.7)	187	(94.4)	0.033 (0.020; 0.047)
Delayed	37	(22.7)	32	(14.3)	11	(5.6)	**-0.213 (-0.307; -0.124)**
**Hospital complexity**							
Low	26	(16.0)	40	(17.9)	31	(15.7)	-0.004 (-0.081; 0.073)
Medium	47	(28.8)	77	(34.4)	85	(42.9)	**0.068 (0.021; 0.115)**
High	90	(55.2)	107	(47.8)	82	(41.4)	**-0.048 (-0.084;-0.012)**
**Total** ^**1**^	163	(17.1)	224	(25.3)	198	(22.8)	**0.044 (0.016; 0.073)**

*β is the slope of the log-binomial model used to measure the temporal trend; 95% CI: 95% Confidence Interval; In bold, statistically significant; ^1^Percentages were calculated taking into account the total number of mastectomized patients in the respective years.

## Discussion

According to the information obtained from the HDD, more than 80% of invasive BC cancer cases diagnosed during the study period underwent breast surgery. These percentages are in accordance with reports from previously published studies in European countries such as Ireland [21] (84% for the period 2004–2008), the United Kingdom (72% in the year 2006) [22] and Switzerland (over 90% for the period 2003–2005) [23].

No surgical interventions were detected for about 17% of the patients in our study. The fact that 60% of these cases presented with a local stage at diagnosis (when surgery is recommended by clinical practice guidelines) [10] could mean that some of these women underwent surgery in private centers before being attended in the public system. This suggests an under-detection of surgical cases in our study. If we add this group of patients to the total surgical cases we did detect, our percentage would probably be close to 90%, similar to those reported in other series in the same time period [21, 23].

Furthermore, the higher percentage of advanced tumor stages among non-surgical, compared to surgical, patient groups, suggests that the tumor stage collected by using a simplification of the Disease Staging method [16] adequately classified those patients considered as non-surgical [24]. In fact, non-surgical, advanced disease stage cases represent about 7.5% of all the breast cancers in our study, similar to the percentage published by other authors [25] and to the results obtained in a BC cancer study carried out by the population cancer registry in the Catalonian province of Girona (6.5% of women were diagnosed at stage IV in 2005, unpublished data).

On the other hand, 82% of our patients presented with local BC. Although this figure seems compatible with the wide coverage of BC screening programs and with the high survival (about 85% at 5 years) of BC patients in Catalonia, it might be overestimated. In the Girona registry, women with stage I-IIB represented just 73% of the total (unpublished data). This discrepancy could be explained by the fact that we discarded the ICD-9-CM code 196.3 (involvement of lymph nodes in the axilla and upper extremity) for local stage classification.

### Breast-conserving surgery versus mastectomy

The percentage of women with BC treated by conservative surgery in Catalonia rose significantly between 2005 and 2011, mainly in the 50–69 year-old age group. These figures (74% in the year 2011) are consistent with other studies in Spain [26, 27] and worldwide [23, 28–30], where reports ranged from 55% to 69%. On the other hand, the United States (US) and Japan show a certain underutilization of this procedure, with percentages ranging from 51% to 59% in the former [31, 32] and just 36% in the latter [33]. Differences in the survey periods, the patient selection criteria, the methods used, the screening programs and the national health policies could all play a role in explaining the differences in these results.

The rise of conservative surgery among regional stage BC patients in Catalonia could be explained by the administration of adjuvant treatment, mainly post-surgical radiation therapy. Other relevant data found in this study shows a generalization of conservative surgery in most Catalonian health centers, with a rising preference for this treatment modality in hospitals with medium and low complexity. The spread of conservative treatment favors equity, regardless of the geographic area where patients live.

As noted by other authors [34, 35], removal of lymphatic structures has increased significantly among patients treated with BCS, with a rise in simple lymph node excision. This finding is closely related to the implementation of sentinel lymph node biopsy (SLNB), which spread in Catalonia over the course of the study period.

In terms of mastectomy, our results for 2005–2011 showed a significant decrease among women treated surgically, in line with data from the European eusomaDB database [36], but in contrast to the trend in the US between 2004 and 2008 [37, 38]. Moreover, mastectomy rates in the Catalonian population were slightly lower than those reported in most European countries [30, 32], Canada [39], the US [32, 37] and Japan [33]. This could suggest a slight underestimation in our study, due to a failure to consider those patients initially treated with conservative surgery who received a mastectomy during a 12-month follow-up period. It should also be noted that (as in our study) most of the cases involved in all these studies were in early breast cancer stages (0, I and II).

An upward trend in mastectomy rates among women under 50 years of age is consistent with other studies [38, 39], and may be explained by several factors, including the higher stage disease at diagnosis, the presence of more biologically aggressive tumors, the higher risk of recurrence over the patients’ lifetime, the availability of immediate breast reconstructive surgery, increasing the aesthetic requirements, patient preference and other intangible factors.

In accordance with other series, patients treated with radical surgery are older and have greater comorbidity compared to those treated conservatively [27, 32].

### Reconstructive surgery after mastectomy

Worldwide, post-mastectomy reconstruction rates have increased over the last decade due to a number of factors, but the procedure is still performed only in a minority of patients. The relatively young age of patients, the local stage of their tumors, the null or low comorbidity, and patient access to more specialized health centers are all recognized as limiting factors in breast reconstruction [40, 41].

In our case, there was a considerable and general increase in reconstructive surgery during the seven-year period, similar to the rise observed in England [42], and in contrast to the trends reported in Canada [39, 40] and Australia [41] (historically low), and in the US [43, 44] (where rates are higher than ours). The rise of breast reconstruction in Catalonia occurred basically due to immediate reconstructive surgery, because delayed breast reconstruction dropped significantly in the study period.

### Methodological strengths and limitations

Our study has several strengths that should be considered. First, the starting point was, the HDD, a population-based information source that has been proved valuable and reliable to carry out clinical and epidemiological studies. Second, the use of ASEDAT software (previously validated) [12], allowed us to identify patients from the first episode of breast cancer care, essentially equivalent to the incidence date. The number of incident breast cancer cases in 2008 in Catalonia (n = 3849) was similar to the estimated number from the Catalonian population-based cancer registries (n = 3907) [8], reinforcing the validity of the methodology used. Third, breast cancer is basically a surgical disease that requires hospitalization, a fact which favors the availability of comprehensive information for primary and reconstructive surgery, because cases are followed for a minimum of 12 and a maximum of 24 months. Finally, information about the application of primary and secondary ICD-9-CM diagnosis and procedures codes allowed us to determine comorbidity and estimate cancer stage, in addition to knowing the surgical techniques implemented.

As for the limitations, we should mention firstly the intrinsic weaknesses of the study design. This is a descriptive study in a database used by Catalonian hospitals, so there could be some variability in coding practices between centers and professionals and the ICD-9-CM changes that happened over the study. For instance, we found some specific deficiencies, such as the lack of any specific ICD-9-CM codes for the most recent techniques, such as SLNB, which prevented us from assessing the impact of its implementation simultaneously with the lymph node dissection.

Secondly, the underestimation of surgical cases by the lack of information on discharges in private hospitals. Thirdly, the lack of relevant information for tumor stage in the HDD, although we tried to mitigate this problem by creating a proxy for the disease stage based on the ICD-9-CM (Additional file 1: Table S2a) [16, 17]. Despite not having been validated in a sample of patient’s medical records, as we mentioned previously, this method seems to have properly delimited the advanced neoplastic disease but overestimated local disease by about 10%. Our results, agree with some validation studies [17, 45, 46], suggests caution when interpreting the results, given the likelihood of overestimating local stages versus advanced stages.

Despite all the aforementioned limitations, this study may be considered an interesting methodological contribution to automated studies of quality indicators from clinical and administrative databases in Catalonia, with regard to surgical procedures for invasive breast cancer.

## Conclusions

In summary, the use of HDD linked to the ASEDAT software allowed us to estimate the incidence and to evaluate breast cancer surgical treatment in Catalonia. Around 80% of women diagnosed with breast cancer over the study periods were treated by conservative surgery. This trend seems to be consolidating in women aged 50–69 and corresponds to the pattern observed in most other European countries. The increase of simple lymph node removal in surgical patients suggests an increase in the practice of sentinel lymph node biopsy. Among the mastectomized patients, immediate breast reconstructions have risen significantly from 2005 to 2011.

## Electronic supplementary material

Additional file 1: Table S1: Contains the ICD-9-CM 6th edition codes associated with different types of surgery in breast cancer; **Table S2a** contains the staging classification codes from ICD-9-CM; **Table S2b** contains the ICD-9-CM codes related to the presence of metastases. (PDF 55 KB)

## Abbreviations

BC: Breast cancer
BCS: Breast conserving surgery
HDD: Hospital discharge database
ASEDAT: Analysis, selection and extraction of tumor data
ICD-9-CM: International classification of diseases, 9^th^ revision, clinical modification
CCI: Charlson comorbidity index
AJCC: American Joint Committee on Cancer
APC: Annual percent change
CI: Confidence interval
US: United States
SLNB: Sentinel lymph node biopsy.
