# Supplementary material for: Trends in the surgical procedures of women with incident breast cancer in Catalonia, Spain, over a 7-year period (2005–2011)
Source: BMC Res Notes. 2014 Sep 1;7:587. doi: 10.1186/1756-0500-7-587 (PMC4165913; doi:10.1186/1756-0500-7-587)
Supplement: Supplementary file 1 — Additional file 1: Table S1: Contains the ICD-9-CM 6th edition codes associated with different types of surgery in breast cancer; Table S2a contains the staging classification codes from ICD-9-CM; Table S2b contains the ICD-9-CM codes related to the presence of metastases. (PDF 55 KB) [file 13104_2014_3128_MOESM1_ESM.pdf]

Table S1 ICD-9-CM 6th edition codes, used for breast cancer surgery classification

ICD-9-CM, 6th edition	Breast surgery	Surgical technique	Surgery type
85.20<br>85.21<br>85.22<br>85.23<br>85.24<br>85.25	Excision/destruction of breast tissue, not otherwise specified (NOS)<br>Local excision of lesion of breast<br>Breast quadrantectomy<br>Subtotal mastectomy<br>Excision of ectopic breast tissue<br>Excision of nipple	Lumpectomy / Partial Mastectomy	Conservative Surgery
85.33 - 85.34<br>85.35 - 85.36	Unilateral subcutaneous mammectomy with synchronous implant / Other unilateral subcutaneous mammectomy<br>Bilateral subcutaneous mammectomy with synchronous implant / Other bilateral subcutaneous mammectomy	Subcutaneous Mastectomy	Radical Surgery
85.41 - 85.42<br>85.43 - 85.44	Unilateral simple mastectomy / Bilateral simple mastectomy<br>Unilateral extended simple mastectomy / Bilateral extended simple mastectomy	Simple Mastectomy	
85.45 - 85.46<br>85.47 - 85.48	Unilateral radical mastectomy / Bilateral radical mastectomy<br>Unilateral extended radical mastectomy / Bilateral extended radical mastectomy	Radical Mastectomy	
	**Excision of lymphatic structure**		**Type of excision**
40.11<br>40.21 - 40.23; 40.29;<br>40.3	Biopsy of lymphatic structure<br>Excision of deep cervical lymph node, internal mammary lymph node or axillary lymph node / Simple excision of other lymphatic structure<br>Regional lymph node excision		Simple
40.40-40.42;40.50;<br>40.51; 40.59	Radical neck dissection, NOS, unilateral and bilateral / Radical excision of lymph nodes NOS;<br>Radical excision of axillary lymph nodes; Radical excision of other lymph nodes		Radical
	**Reconstructive surgery**	**Type of mastoplasty**	
85.33; 85.35<br>85.53 - 85.54	Unilateral subcutaneous mammectomy with synchronous implant / Bilateral subcutaneous mammectomy with synchronous implant<br>Unilateral breast implant / Bilateral breast implant	Augmentation	Implants
85.51 - 85.52<br>85.95	Unilateral injection into breast for augmentation / Bilateral Injection into breast for augmentation<br>Insertion of breast. Tissue expander		Injection / Expander
85.82 - 85.85	Split -thickness graft to breast / Muscle flap graft to breast		Flap
85.50	Augmentation mammoplasty NOS		Not specified
85.31 - 85.32<br>85.94<br>85.96	Unilateral reduction mammoplasty / Bilateral reduction mammoplasty<br>Removal of implant of breast<br>Removal of breast tissue expander	Reduction	
85.7<br>85.81<br>85.86 - 85.87<br>85.89<br>85.93<br>85.99	Total reconstruction of breast, NOS<br>Suture of laceration of breast<br>Transposition of nipple / Other repair or reconstruction of nipple<br>Other mammoplasty<br>Revision of implant of breast<br>Other	Others	

85.33 and 85.35 ICD-9-CM codes corresponds simultaneously to invasive surgery and reconstruction

Table S2a Breast cancer staging from ICD-9-CM codes

	ICD-9-CM 6th edition code	Description	Stage	AJCC stage
**(1)**	174.0 - 174.9	Malignant neoplasm of female breast	Local	I
**(2)**	**(1)** AND 196.3	Secondary and unspecified malignant neoplasm of lymph nodes of axilla and upper limb	Regional	II
**(3)**	**[(1) and/or (2)]** AND [196.0-196.2, 196.5-196.9, 197.0-197.8, 198.0-198.8]	Secondary malignant neoplasm	Distance	III-IV

Source**:** Adapted from: 1) *Disease Staging: Clinical Criteria*. Version 5.22. Thomson Medstat, 2003; Available at; http://www.hcup-us.ahrq.gov/db/nation/nis/Disease%20Staging%20V5.22%20Clinical%20Criteria.pdf and 2) Yuen E, Louis D, Cisbani L et al. Using administrative data to identify and stage breast cancer cases: implications for assessing quality of care. *Tumori* 2011; 97 (4): 428-35**.**

Table S2b ICD-9-CM 6th edition codes related with the presence of metastasis

ICD-9-CM code	Description
196.0	Secondary and unspecified malignant neoplasm of lymph nodes of head, face, and neck
196.1	Secondary and unspecified malignant neoplasm of intrathoracic lymph nodes
196.2	Secondary and unspecified malignant neoplasm of intra-abdominal lymph nodes
196.5	Secondary and unspecified malignant neoplasm of lymph nodes of inguinal region and lower limb
196.6	Secondary and unspecified malignant neoplasm of intrapelvic lymph nodes
196.8	Secondary and unspecified malignant neoplasm of lymph nodes of multiple sites
196.9	Secondary and unspecified malignant neoplasm of lymph nodes, site unspecified
197.0	Secondary malignant neoplasm of lung
197.1	Secondary malignant neoplasm of mediastinum
197.2	Secondary malignant neoplasm of pleura
197.3	Secondary malignant neoplasm of other respiratory organs
197.4	Secondary malignant neoplasm of small intestine including duodenum
197.5	Secondary malignant neoplasm of large intestine and rectum
197.7	Malignant neoplasm of liver, secondary
197.8	Secondary malignant neoplasm of other digestive organs and spleen
198.0	Secondary malignant neoplasm of kidney
198.1	Secondary malignant neoplasm of other urinary organs
198.2	Secondary malignant neoplasm of skin
198.3	Secondary malignant neoplasm of brain and spinal cord
198.4	Secondary malignant neoplasm of other parts of nervous system
198.5	Secondary malignant neoplasm of bone and bone marrow
198.6	Secondary malignant neoplasm of ovary
198.7	Secondary malignant neoplasm of adrenal gland
198.81	Secondary malignant neoplasm of breast
198.82	Secondary malignant neoplasm of genital organs
198.89	Secondary malignant neoplasm of other specified sites
